# Paraquat-induced cholesterol biosynthesis proteins dysregulation in human brain microvascular endothelial cells

**DOI:** 10.1038/s41598-021-97175-w

**Published:** 2021-09-13

**Authors:** Vujić Tatjana, Schvartz Domitille, Sanchez Jean-Charles

**Affiliations:** 1 Department of Medicine, Faculty of Medicine, University of Geneva, Geneva, Switzerland; 2 Swiss Center for Applied Human Toxicology, Geneva, Switzerland

**Keywords:** Proteomics, Molecular neuroscience

## Abstract

Despite Paraquat (PQ) being banned in several countries, it is still one of the most commonly used herbicides in agriculture. This compound is known to induce damaging effects on human and animal brain cells by generating Reactive Oxygen Species (ROS). However, there is few evidence of PQ effect on Human Brain Microvascular Endothelial Cells (HBMECs), one of the major component of the Blood–Brain Barrier (BBB). The present study aimed at unraveling biological mechanisms associated to the exposure of 1, 10 and 100 µM of PQ for 24 h on HBMECs. High-throughput mass spectrometry-based proteomics using data-independent acquisition (DIA) was applied. Biological pathway enrichment and cellular assays such as mitochondrial respiration and cholesterol level were performed to verify proteomics results. A total of 3753 proteins were quantified out of which 419 were significantly modulated by paraquat exposure. Biological pathway enrichment revealed the ubiquinone metabolism, a pathway directly linked to mitochondrial complex I proteins, confirming the well-known mechanism of PQ inducing oxidative stress. Additionally, this study also described the cholesterol biosynthesis modulation on HBMECs not yet described. In conclusion, our data indicate the toxic effect of PQ on HBMECs by downregulating proteins involved in mitochondrial complex I and cholesterol pathways.

## Introduction

Epidemiological and toxicological studies have reported an association between environmental toxicant exposure and neurodegenerative diseases^1–5^. Among these environmental toxicants, paraquat (PQ) is one of the most widely used herbicide in many parts of the world. Nevertheless, PQ is also known for its acute toxicity and chronic effects^3,6^. Agricultural workers and farmers, who are not sufficiently protected, are still applying this chemical and increasing risk of developing adverse health effects such as respiratory system default, reproductive problems or increasing about twofold their risk of developing Parkinson’s disease^4,5,7–13^. Because of these evidences, PQ was banned by the European Court of First Instance in several countries, mostly in the European Union countries^14^. However, despite the known public health issues of PQ, it is still applied by millions of agricultural workers in about 100 countries, mainly the global south countries but also by the United States. Moreover, data from US Geological Survey’s Pesticide National Synthesis Project pointed out that use of PQ doubled over the past decade.

The toxic mode of action of PQ is to induce oxidative stress through the generation of Reactive Oxygen Species (ROS), creating a cell redox imbalance^1,15,16^. In this redox relation, interaction of PQ with mitochondria remains an important and one of the most described aspect of its toxicity, particularly in brain^17,18^. Mitochondrial complex I and III activities are the two main complexes from the electron chain transport impacted by PQ exposure. Their alteration lead to the inhibition and dysfunction of mitochondrial function^17–19^. Despite the presence of the blood–brain barrier (BBB), a unique anatomical and physiological barrier between the bloodstream and the extracellular space of the brain, it was demonstrated that PQ can be transported into the brain by neutral amino acid transport system^6,20^. This toxin is also eliminated much more slowly in the brain than any another organ such as the liver, suggesting longer-lasting effects in the brain.

HBMECs are different from other endothelial cells due to their unique phenotype^21,22^. They possess specialized junctional complexes such as tight, adherens or gap junctions. These junctions are able to maintain the BBB integrity^23,24^ by connecting HBMECs together. Thereby, they prevent compounds from paracellular diffusion across the BBB^25–27^. The existence of specific metabolic transporters (e.g. solute carrier and ATP-binding cassette family) also control the movement of nutrients, ions, toxins or xenobiotics across the endothelia^21^. Moreover, HBMECs are determined by intimate contact with other members of the neurovascular unit (NVU) such as neurons, astrocytes, microglia, pericytes and extracellular matrix molecules^27^. These complex interactions contribute to the dynamic regulation of microvascular permeability and regulate the function of HBMECs during normal and abnormal BBB activity^28–30^, explaining their importance in brain research.

Since environmental toxicant are important risk factors of neurodegeneration, it is crucial to put forward mechanisms by which they cause neurotoxicity. The aim of this study was to identify detrimental mechanisms caused by PQ on HBMECs. Data-independent acquisition mass spectrometry (DIA-MS) was applied to explore proteome profiles and biological pathways impacted by PQ at 1, 10 and 100 µM for 24 h and to demonstrate its role as an oxidative stress inducer on HMBECs. Proteomics and biological pathway enrichment analysis provide evidence of the modulation of a well-known pathway—the ubiquinone metabolism—linked to the mitochondrial complex I proteins. More is being learned about the effects of exposure to PQ on HBMECs as results provide novel insight on the alteration of cholesterol biosynthesis.

## Results

### MTS proliferation and LDH cytotoxicity assays of human brain microvascular endothelial cells exposed to paraquat

PQ-induced toxicity was evaluated in HBMECs for 24 h. MTS proliferation assay indicated that PQ concentrations above 100 µM were significantly decreasing cell proliferation (Supplementary Fig. S1). Regarding cytotoxicity, PQ concentration above 100 µM was significantly increasing cellular death indicating its toxic effect (Supplementary Fig. S1). These results suggested that PQ concentration at 0.1, 1, 10 and 100 µM did not affect cell proliferation and had no cytotoxic effect on HBMECs. Moreover, other in vitro and in vivo studies applying PQ demonstrated that concentration up to 100 µM can be used^31–33^.

### Proteomics and pathway enrichment analysis of human brain microvascular endothelial cells after paraquat exposure

**Figure 1:**
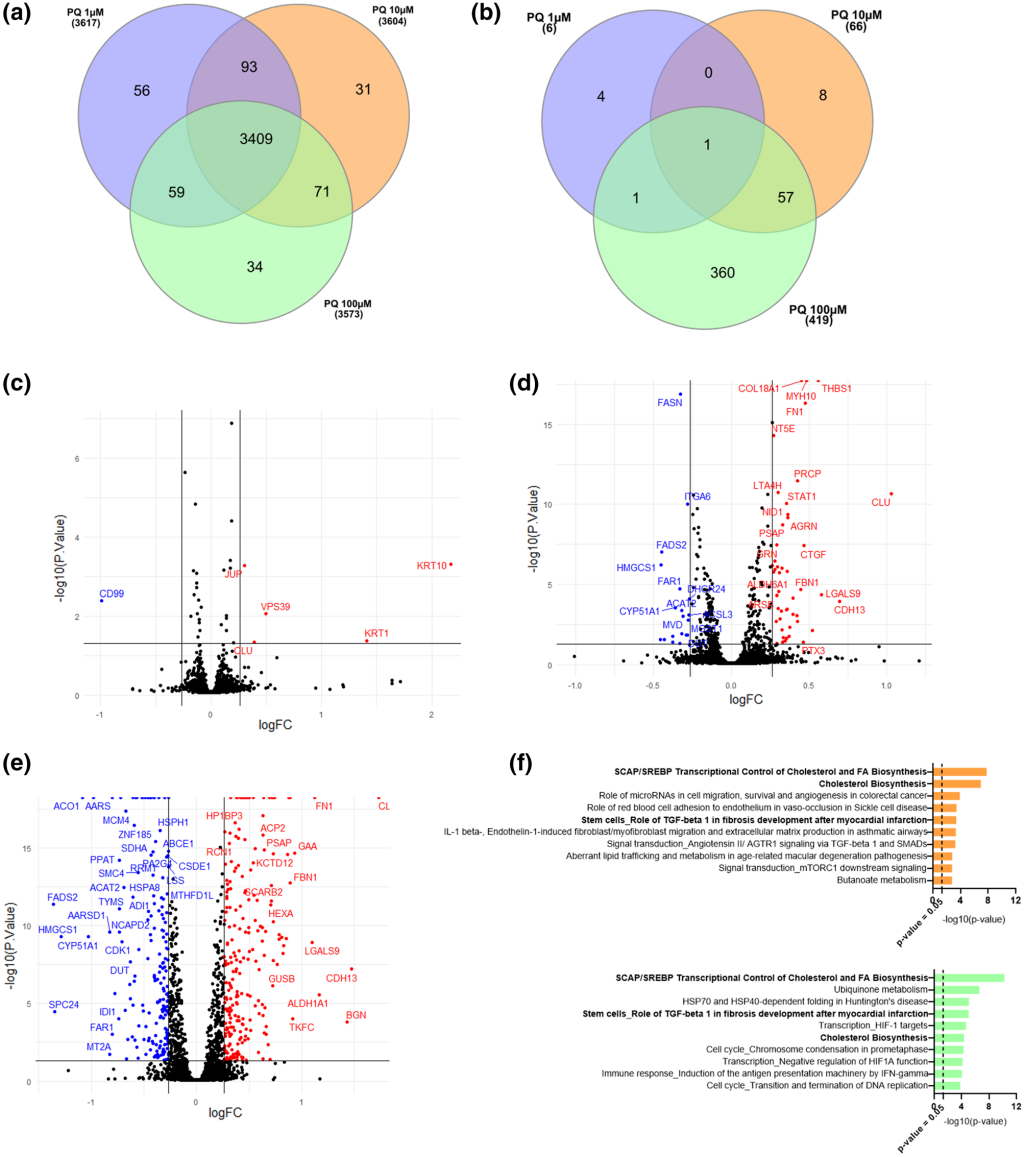
(**a**–**f**) Proteomics data and enrichment pathway results of PQ-treated HBMECs at 1 µM, 10 µM and 100 µM for 24 h. (**a**) Venn diagram displaying a comparison of total quantified proteins in HBMECs after PQ concentrations at 1, 10 and 100 µM. (**b**) Venn diagram displaying a comparison of total DEPs in HBMECs after PQ concentrations at 1, 10 and 100 µM. (**c**–**e**) Volcano plots displaying the distribution of all proteins after PQ concentrations at 1 µM (**c**), 10 µM (**d**) and 100 µM (**e**) in HBMECs. Downregulated proteins are in blue. Upregulated proteins are in red. X axis corresponds to log_2_(FC), Y axis corresponds to − log_10_(*p*-value). (**f**) Top ten of pathway maps enriched by MetaCore software from the lists of DEPs (FC > 1.2, *p*-value ≤ 0.05, *N* = 3) after PQ concentrations at 1 µM (purple), 10 µM (orange) and 100 µM (green) for 24 h. The top 10 pathways for each condition are represented in the graph. X axis corresponds to − log_10_(*p*-value), Y axis corresponds to the name of biological pathways and the dashed line represents the enrichment *p*-value cut-off of 0.05.

To further elucidate PQ-induced mechanisms, Data Independent Acquisition (DIA)-based proteomics analysis was applied. More than 3500 proteins were quantified for PQ-exposed HMBECs at 1, 10 and 100 µM, respectively (Fig. 1a).

Only six proteins were differentially expressed in HBMECs exposed to PQ at 1 µM versus untreated control (Fig. 1b and Supplementary Table S1). Sixty-six proteins were modified by PQ concentration at 10 µM (Fig. 1b and Supplementary Table S2). Finally, 419 proteins were differentially expressed for PQ at 100 µM (Fig. 1b and Supplementary Table S3). These results indicated that the number of differentially expressed proteins (DEPs) is associated to a dose–response manner (Fig. 1c–e). To analyze PQ-effect on HMBECs at the biological pathway level, an enrichment analysis was performed in Metacore software. DEP lists from proteomics experiments were used (Supplementary Tables S1, S2 and S3). Due to the very low number of DEPs in HBMECs exposed to PQ at 1 µM versus untreated control, enrichment pathway was not considered. Pathways enrichment of HBMECs exposed to PQ at 10 µM show highly significant enrichment *p*-value for the two biological pathways—“SCAP/SREBP Transcriptional Control of Cholesterol and FA Biosynthesis” and ”Cholesterol Biosynthesis ” (*p* value of 1.61 × 10^−08^ and 1.11 × 10^−07^, respectively) (Fig. 1f). Both of these impacted biological pathways were also observed in the top ten of the pathways modified in HMBECs exposed to PQ at 100 µM—“SCAP/SREBP Transcriptional Control of Cholesterol and FA Biosynthesis” (*p* value = 6.83 × 10^−11^) and ”Cholesterol Biosynthesis ” (*p* value = 4.93 × 10^−05^) (Fig. 1f). However, the second most affected pathway after PQ exposure at 100 µM revealed to be the “Ubiquinone Metabolism” (*p* value = 2.90 × 10^−07^) (Fig. 1f).

### Ubiquinone metabolism modulation after paraquat exposure on human brain microvascular endothelial cells

**Figure 2:**
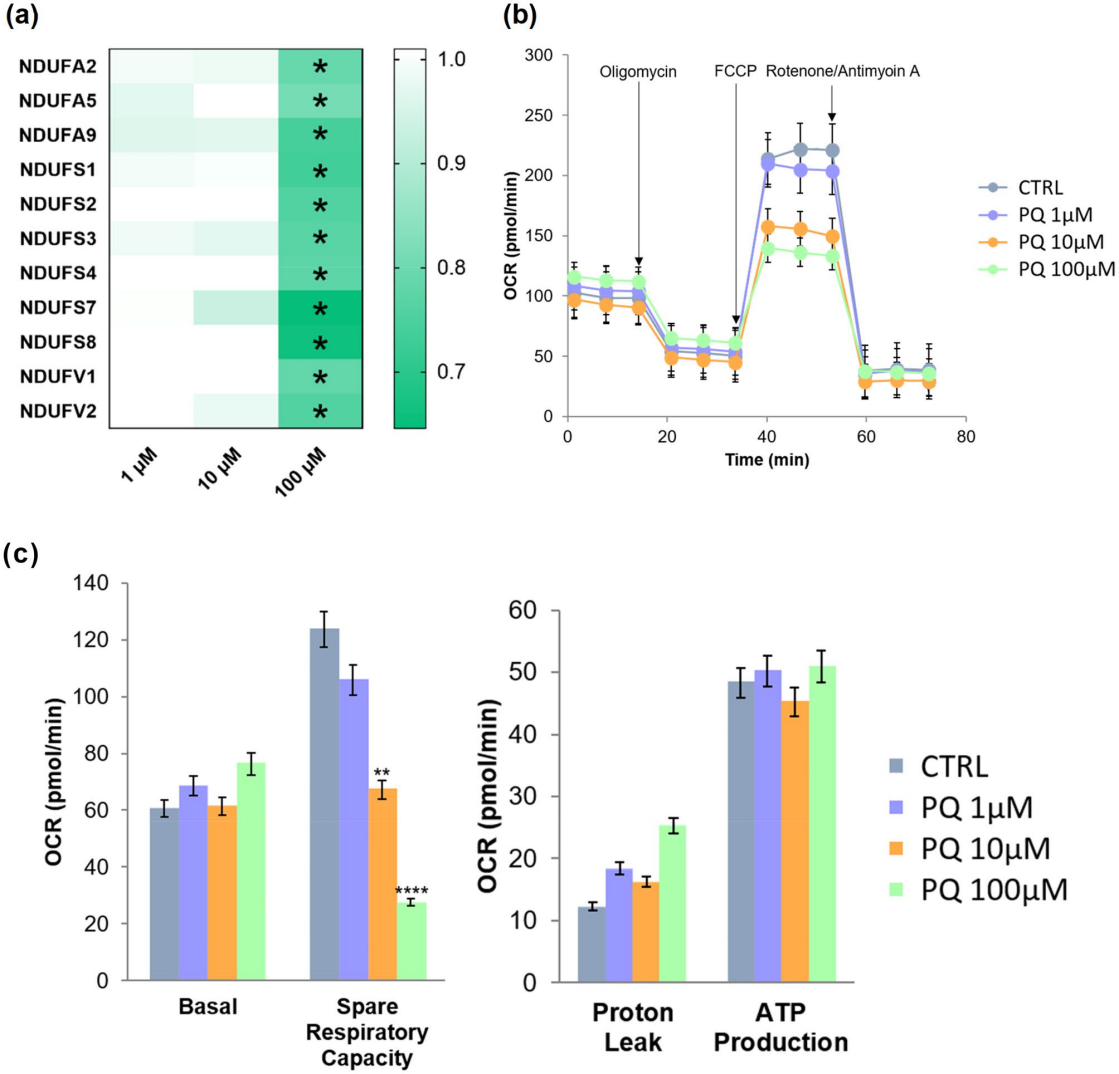
(**a**–**c**) Mitochondrial dysfunction of PQ-treated HBMECs. (**a**) Heat map of NADH-ubiquinone proteins obtained by MS and altered by PQ exposure. Stars indicate that protein level was modified in HBMECs exposed to PQ for 24 h (LFDR ≤ 0.05; |FC|> 1.2). (**b**) Time-course experiment to assess mitochondrial stress in HBMECs using different PQ concentrations (1, 10 and 100 μM) and control (water). (**c**) Basal respiration, spare respiratory capacity, proton leak and ATP production are represented for each concentration. Data are represented as means ± SD of three biological replicates. ** corresponds to *p*-value ≤ 0.01 and **** corresponds to *p*-value ≤ 0.0001.

Proteomics results as well as pathway enrichment analysis of PQ-exposed HBMECs at 100 µM highlighted a highly altered biological process linked to oxidative stress; the ubiquinone metabolism (Fig. 1). This modulated pathway is implicated in mitochondrial dysfunction, a toxic effect of PQ widely studied mostly in the rat brain^15,18,34^. Based on pathway enrichment results, the heat map illustrated proteins involved in the ubiquinone metabolism (Fig. 2a).

To verify this observation, as NADH-ubiquinone oxidoreductase proteins are involved in the first step of mitochondrial respiration^35^, a mitochondrial dynamics and bioenergetics assay of PQ-exposed HBMECs was performed. Mitochondrial function assay showed a significant decrease of maximal respiration and spare respiratory capacity for PQ-treated HBMECS at 10 µM and 100 µM (Fig. 2b,c). These results combined to quantitative proteomic results and biological pathway enrichment analysis confirmed that HBMECs exposed to the highest PQ concentration are more susceptible to trigger an oxidative stress-related mechanism.

### Cholesterol biosynthesis modulation induced by paraquat on human brain microvascular endothelial cells

**Figure 3:**
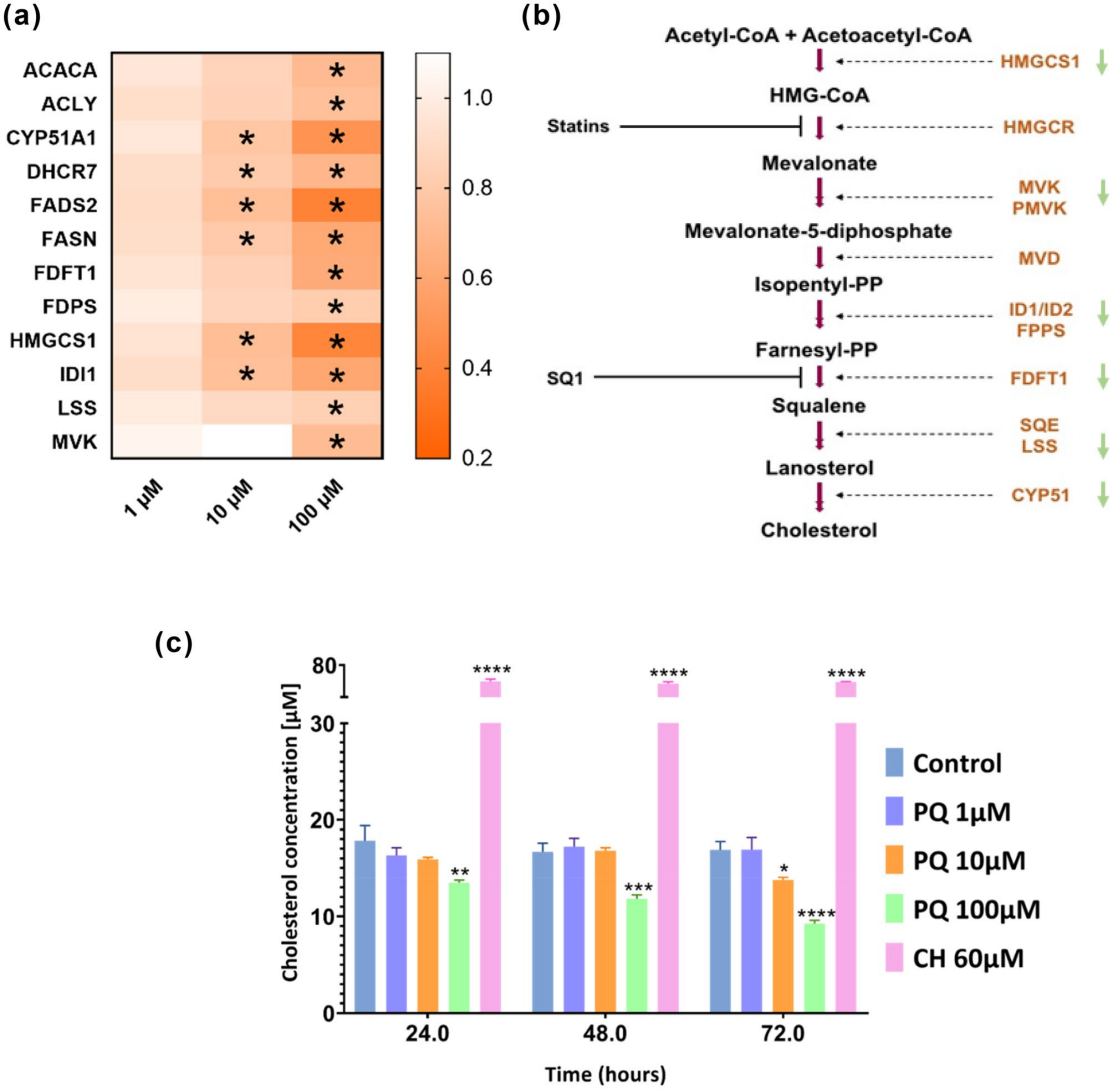
Cholesterol biosynthesis modulation in HBMECs after PQ exposure. (**a**) Heat map of cholesterol proteins obtained by MS and altered by PQ exposure. Stars indicate that protein level was modified in HBMECs exposed to PQ for 24 h (LFDR ≤ 0.05; |FC|> 1.2). Protein fold-change (FC) were displayed as colours ranging from orange to white as shown in the key. (**b**) Representation of the cholesterol biosynthesis key steps. Arrows indicate that protein was decreasing in HBMECs exposed to PQ for 24 h (LFDR ≤ 0.05; |FC| > 1.2). Cholesterol measurement of HBMECs exposed to PQ for 24 h. (**c**) Time-course run of cholesterol measurement using different PQ concentrations (1, 10 and 100 μM), control (water) and positive control (cholesterol 60 μM). Data are represented as means ± SD of three biological replicates. * corresponds to *p*-value ≤ 0.05, ** corresponds to *p*-value ≤ 0.01, *** corresponds to *p*-value ≤ 0,001 and **** corresponds to *p*-value ≤ 0.0001.

Proteomics results as well as pathway enrichment analysis (Fig. 1) suggested that cholesterol biosynthesis is highly altered in HBMECs exposed to PQ at 10 and 100 µM. Fifty-seven DEPs were shared between PQ concentrations at 10 and 100 µM (Fig. 1b). Of those 57 DEPs, a cluster was retrieved as the cholesterol and lipid biosynthetic process (FASN, ACSL3, FADS2, HMGSCS1, DHCR7, IDI1, DHCR24, CYP51A1, MVD, ACAT2, FAR1) (Fig. 3a). The heat map demonstrated that key proteins involved in cholesterol biosynthesis were significantly decreasing as PQ concentration is increasing (Fig. 3a). These crucial proteins were involved in every step of the cholesterol biosynthesis as demonstrated in Fig. 3b, meaning that there is a global impact on cholesterol biosynthesis in HBMECs due to PQ exposure (starting at 10 µM of PQ).

To verify cholesterol biosynthesis modulation after PQ exposure, a cellular assay measuring cholesterol level was performed with three different PQ concentrations (1, 10 and 100 µM) for 24 h, 48 h and 72 h. The cholesterol assay revealed a significant decrease in cholesterol level at 10 µM of PQ for 72 h as well as at 100 µM for 24 h, 48 h and 72 h (Fig. 3c). These results combined to proteomics and enrichment pathway analysis (Fig. 1) supported the hypothesis that HBMECs exposed to PQ could affect the cellular cholesterol biosynthesis.

## Discussion

Environmental xenobiotics are still a public concern due to their adverse effects. Indeed, exposure to paraquat (PQ), a non-selective herbicide, has shown some negative effects on agricultural workers such as reduced lung function, skin burn or irritation and eye damage^9,36,37^. In addition, epidemiological and toxicological studies have also demonstrated a link between paraquat exposure and an increasing risk to develop neurodegenerative diseases^1–3,38,39^. PQ acts by generating superoxide radicals resulting in lipid peroxidation of cellular membranes^40^. In studies conducted on animal and human brain, it has largely been documented that PQ is able to inhibit the electron transport chain within mitochondria leading to the production of reactive oxygen species (ROS)^15,16,18,34,41,42^. However, even if PQ toxicity was verified in plethora of research, its use is strongly widespread and often under unsafe and no restricted conditions.

Moreover, many studies have shown that ROS generation is damaging brain cells such as neurons or astrocytes but consequences of PQ exposure in human brain microvascular endothelial cells are still not investigated. This study aimed to explore and give an insight to biological pathways impacted by PQ exposure by using a proteomics quantitative MS-based strategy and enrichment pathway analysis.

PQ concentrations of 1, 10 and 100 µM were selected according to our results from cytotoxicity and proliferation assays and based on several in vitro and in vivo studies using PQ^31–33,43,44^. Indeed, a study using astrocytes and neurons has reported that PQ elicit significant effect at 100 µM after 24 h exposure, supporting PQ concentration chosen in our study as well as the short exposure time of 24 h^31^. In another research using mouse brain, they selected PQ concentration of 100 µM as the highest one^32^. However, it is worth noticing that PQ-exposed astrocytes concentration used in the study of Zheng et al. was between 2 to 8-fold higher than in our research^45^. With this last study, we notice that PQ concentration is cell-type dependent and we highlight the need to be confirmed by cellular assays. Additionally, results from a recent paper from Yuan et al*.* described that blood concentration of patients after paraquat acute poisoning ranged from 0.10 to 20.62 μg/mL, which correspond to 0.38–80 µM. Nonetheless, our study was performed on a primary cell monolayer culture meaning that interaction effect with other cell types of the BBB (i.e.: glial cells, neurons) is not considered. This interaction may strongly modify and influence brain endothelial cells phenotype as well as PQ concentration to use.

As described in many studies, PQ is causing mitochondrial dysfunction due to the cytotoxic molecule generation such as nitric oxide radicals or other ROS^18,34,42,46–50^. Similarly, our proteomics results has shown 11 altered proteins involved in the mitochondrial respiratory complex I (NADH-ubiquinone oxidoreductase). Pathway enrichment analysis as well as respirometric assay of mitochondria biogenesis verified these results by indicating an altered pathway linked to complex I of mitochondrial respiration and a statistically significant decrease of mitochondrial function in a dose-dependent manner. These results are consistent with previous studies supporting a deleterious effect of PQ on mitochondria in other cell types^18,34,42,46–49^. They are also in line with three studies displaying an association with dysfunction or inhibition of mitochondrial complex I activities^5,47,51^. Thereby, reduction or alteration in ubiquinone synthesis could impair TCA cycle and mitochondrial respiration, which might change redox environment causing mitochondrial dysfunction. Our findings led to support that PQ is inducing mitochondrial dysfunction also on human brain microvascular endothelial cells.

Furthermore, our proteomics data analysis highlighted the modulation of the cholesterol biosynthesis pathway in PQ-treated HBMECs. Cholesterol biosynthesis, originated from the mevalonate pathway, is vital to ensure fundamental cellular processes^52–54^. Indeed, this sterol is required to reinforce and organize cell membrane as well as to maintain and form lipid rafts^52–54^. Additionally, cholesterol has an essential role by acting as a precursor of steroid hormones, bile acids and oxysterols, and as a regulator of cell signalling^52–54^. In the brain, cholesterol is synthetized in situ*,* as the plasma lipoproteins are not crossing an intact BBB^53,55,56^. The first step of mevalonate pathway resulting to the cholesterol synthesis is executed by the transferase, 3-hydroxy-3-methylglutaryl-CoA synthase 1 (HMGCS)^57,58^. This critical cholesterol biosynthesis enzyme is responsible of the acetyl coenzyme A condensation with acetoacetyl coenzyme A to produce HMG-CoA by a catalytic process^57,58^. In the present study, this protein was found to be the most significantly decreased, pointing up the first failure in the cholesterol biosynthesis cascade. Smith et al*.* indicated that reduced expression of HMGCS in mice cerebral cortex is linked to a cholesterol downregulation, supporting our observation^59^. The mevalonate kinase (MVK), an enzyme involved in the conversion of the mevalonate into the mevalonate-5-phosphate, is the converting enzyme following that of HMGCR. It was also shown to be significantly downregulated in this cascade. Furthermore, one study had recognized that MVK deficiency could cause inflammatory and CNS disorders^60^, suggesting that cholesterol biosynthesis in HBMECs may be impacted by PQ exposure. The next step of this cholesterol cascade is the isopentenyl-diphosphate Delta-isomerase 1 (IDI1) that catalyzes isopenty-pyrophosphate into farnesyl-pyrophosphate^61^. As shown in our results, IDI1 was significantly decreased by PQ exposure in HBMECs. Some studies have demonstrated that cellular expression of IDI1 could be inhibited by ROS generation, resulting in a diminution of lipophilic molecules formation such as ubiquinone, sterols and terpenoids. This observation is suggesting a similar effect induced by ROS generation of the PQ in our study^62,63^. In addition, a recent study of Tan et al*.* reported a reduction in cholesterol level, as well as a mitochondrial dysfunction on HBMECs by atorvastatin, which is an inhibitor of cholesterol production^64^. Our study has also highlighted a significant decrease of cellular cholesterol in a time- and dose-dependent manner. These results are corroborated with the downregulation of key proteins involved in the cholesterol biosynthesis found by DIA quantification and shown by enrichment pathway analysis. A previous study demonstrated the reduction of cholesterol concentration in PQ-treated astrocytes which might support our hypothesis^45^. These combined observations suggested a PQ-induced toxicity at cellular cholesterol level in brain cells, as astrocytes are tightly in interaction with brain endothelial cells in the BBB. Consequently, dysregulated proteins of the cholesterol cascade and decreased cholesterol level observed in PQ-treated HBMECs could actively affect cell membrane structure and compromise its integrity, by disrupting cellular homeostasis as well as leading to cellular death^65^. Indeed, the brain demands a constant level of cholesterol in physiological conditions, underlying the need to maintain an optimal cholesterol level for energetic metabolism, cell membrane composition as well as myelination^66^. As PQ exposure on HBMECs revealed a decreased level of cholesterol, it can be hypothesized that the previously cited processes may be impaired. To add, a causal relationship was reported with oxidative stress generation and reduction of cellular cholesterol in brain diseases^67^, displaying the potential link between oxidative stress exert by PQ and alteration of the cholesterol metabolism on human brain endothelial cells.

In conclusion, this study underlined that most of the differentially expressed proteins in PQ-treated HBMECs were involved in redox pathways. As expected, mitochondrial dysfunction was denoted after PQ exposure on HBMECs in a dose-dependent manner. Both oxidative stress, demonstrated by affected ubiquinone metabolism and the decrease of mitochondrial maximal respiration, might strongly alter endothelial function by impairing maintenance of the BBB integrity^23,24^.

This study also highlighted for the first time that PQ is able to modify protein level of proteins involved in cholesterol biosynthesis as well as reduce cellular cholesterol level. Modulation of cholesterol biosynthesis may potentially affect cell membrane structure and compromise its integrity, having a direct effect on energetic metabolism or myelinisation process in brain.

Given the ethical complexity to study a toxicant effect on human population, this in vitro research gives novel insights of PQ toxicity as it pointed out cholesterol alteration on HBMECs. This additional biological alteration is reinforcing the need to definitively ban PQ as an herbicide or to investigate for a safer solution for agricultural workers.

## Methods

### Cell culture and treatments

Primary human brain microvascular endothelial cells (ACBRI 367, Cell Systems) were cultured onto a rat tail collagen type I-coated (15 µg/mL, Merck Millipore) plates at 50,000 cells/cm^2^ and maintained in complete endothelial cell growth medium-2 (EGM-2MV BulletKit, Lonza) at 37 °C in a 5% CO_2_ incubator. At 80% of confluence, cells were treated with Paraquat (Sigma Aldrich) (1, 10 and 100 µM) for 24 h. Afterwards, cells were detached with Stempro Accutase (Gibco) and washed three times with ice-cold Phosphate Buffered Saline (PBS, Gibco) and dry-stored at − 80 °C.

### MTS proliferation and LDH cytotoxicity assay

HBMEC were seeded in a 96-well plate (10,000 cells per well) and treated for 24 h with PQ at different concentrations (0.1, 1, 10, 100, 1000 and 5000 µM). Cell proliferation was determined using the MTS assay (CellTiter 96 AQueous One Solution Cell Proliferation Assay, Promega), whereas cytotoxicity was assessed by measuring lactate dehydrogenase (LDH) released using a Pierce LDH cytotoxicity kit (Thermo Scientific). Both the MTS and LDH assays were performed according to the manufacturer’s recommendations.

### Sample preparation for mass spectrometry-based proteomics

Cell pellets were resuspended in 0.1% RapiGest (Waters) and 100 mM TEAB (Sigma Aldrich), sonicated (five cycles of 20 s with breaks on ice), and incubated for 10 min at 80 °C. Samples were then spun down (14,000*g*, 10 min, 4 °C) and the supernatant was recovered. The protein content was measured using Bradford assay (BioRad).

For each sample, 20 μg proteins was reduced using TCEP (final concentration 5 mM, 30 min, 37 °C) (Sigma Aldrich), alkylated using iodoacetamide (final concentration 15 mM, 60 min, RT, in dark condition) (Sigma Aldrich) and digested by an overnight tryptic digestion (w/w ratio 1:50) (Promega). The RapiGest surfactant was cleaved by incubating samples with 0.5% trifluoacetic acid (Sigma Aldrich) (45 min, 37 °C). Samples were then desalted on a C18 reverse phase columns (Harvard Apparatus), peptides were dried under vaccum and subsequently resuspended in 5% ACN 0.1% FA (peptides final concentration of 0.5 μg/μL and spiked with iRT peptide (Biognosys) (1:20)).

### MS data independent acquisition and data analysis

For each sample, the equivalent of 2 μg of peptides were analyzed using Liquid Chromatography-Electrospray ionization-MS/MS (LC–ESI–MS/MS) on an Orbitrap Fusion Lumos Tribrid mass spectrometer (Thermo Fischer Scientific) equipped with an EASY nLC1200 liquid chromatography system (Thermo Fisher Scientific). Peptides were trapped on a 2 cm × 75 μm i.d. PepMap C18 precolumn packed with 3 μm particles and 100 Å pore size. Separation was performed using a 50 cm × 75 μm i.d. PepMap C18 column packed with 2 μm and 100 Å particles and heated at 50 °C. Peptides were separated using a 160 min segmented gradient of 0.1% formic acid (solvent A) and 80% acteonitril 0.1% formic acid (solvent B) (Supplementary Table S4), at a flow rate of 250 nl/min. Data-Independant Acquisition (DIA) was performed with MS1 full scan at a resolution of 60,000 (FWHM) MS1 was acquired in the Orbitrap with an AGC target of 3 × 10^6^, a maximum injection time of 100 ms, a scan range from 400 to 1250 m/z followed by 30 DIA MS2 scan with variable windows. DIA MS2 was performed in the Orbitrap using higher-energy collisional dissociation (HCD) at 30%. AGC target of 2 × 10^6^ and a maximum injection time of 80 ms. The raw DIA MS data were matched against the spectral library following the published protocol^68^.

Protein abundances were exported from Spectronaut. We also used peptides intensities which were exported and analyzed using mapDIA^69^. No further normalization was applied. The following parameters were used: min peptides = 1, max peptides = 10, min correl =  − 1. Min_DE = 0.01, max_DE = 0.99, and experimental_design = independent design. Proteins were considered to have significantly changed in abundance with a LFDR < 0.05 and an absolute fold change (|FC|) > 1.2.

### Biological pathway analysis

The list of differentially expressed proteins from PQ-treated HBMECs (Supplementary Tables S1, S2 and S3) were submitted to MetaCore version 21.2 (Clarivate Analytics) to highlight significantly represented biological pathways. Top 10 biological pathways were selected.

### Mitochondrial function—XF cell mito stress test

Mitochondrial respiration was measured using a XF96 extracellular flux analyzer (Seahorse Bioscience, Agilent). The provided 96 well Agilent Seahorse XF Cell Culture Microplate was coated with a solution of rat tail collagen type I (15 µg/mL, Merck Millipore). HBMECs were seeded at a density of 75,000 cells/well, treated with PQ at 1, 10 and 100 µM and maintained in complete endothelial cell growth medium-2 (EGM-2MV BulletKit, Lonza) at 37 °C in a 5% CO_2_ incubator for 24 h. The sensor cartridge was hydrated with the provided XF Calibrant at 37 °C in a non-CO_2_ incubator overnight. The culture medium was refreshed 1 h prior to the assay using an optimized medium containing complete endothelial cell growth medium-2 (EGM-2MV BulletKit, Lonza) without serum and with HEPES (final concentration 20 mM, Gibco). Microplate and four mitochondrial inhibitor drugs were subsequently loaded to the hydrated cartridge after reached the optimal concentration for each compound according the manufacturer’s protocol. Briefly, oligomycin (final concentration 4 µM, Sigma-Aldrich), FCCP (final concentration 16 µM, Sigma-Aldrich) and rotenone and antimycin A (final concentration 2 µM, Sigma-Aldrich) were loaded to the hydrated cartridge. All the parameters were considered as explained in Smolina et al. paper^70^.

### Cholesterol assay

HBMECs were seeded in a 96 opaque-walled assay plate (40,000 cells per well) and treated for 24 h with PQ at different concentrations (1, 10, 100 µM). Water was used as control and spiked cholesterol at 60 µM as positive control. Cholesterol was measured using a cholesterol kit assay (Cholesterol Assay, Promega). The cholesterol assay was performed according to the manufacturer’s recommendation.

### Statistical analysis

Data are reported as mean ± standard deviation (S.D.). p < 0.05 was considered as statistically significant. Significance is denoted as *p < 0.05, **p < 0.01, ***p < 0.001, ****p < 0.0001. The data were analyzed using multiple t-test comparisons or one-way analysis of variance (ANOVA).

## Supplementary Information

Supplementary Figure S1.

Supplementary Table S1.

Supplementary Table S2.

Supplementary Table S3.

Supplementary Table S4.

## Data Availability

Data are available via ProteomeXchange with identifier PXD026975, username: reviewer_pxd026975@ebi.ac.uk, password: jjlUHMR5.

## Abbreviations

PQ: Paraquat
ROS: Reactive oxygen species
HBMECs: Human brain microvascular endothelial cells
BBB: Blood–brain barrier
NVU: Neurovascular unit
DIA-MS: Data-independent acquisition-mass spectrometry
DIA: Data independent acquisition
MS: Mass spectrometry
DEPs: Differentially expressed proteins
FC: Fold change
LFDR: Local false discovery rate
