# Supplementary material for: Paraquat-induced cholesterol biosynthesis proteins dysregulation in human brain microvascular endothelial cells
Source: Sci Rep. 2021 Sep 13;11:18137. doi: 10.1038/s41598-021-97175-w (PMC8438088; doi:10.1038/s41598-021-97175-w)
Supplement: Supplementary file 1 — Supplementary Figure S1. [file 41598_2021_97175_MOESM1_ESM.docx]

Paraquat-induced cholesterol biosynthesis proteins dysregulation in human brain microvascular endothelial cells

Vujic Tatjana^1,2^, Schvartz Domitille^1,2^ and Sanchez Jean-Charles^1,2^

^1^ Department of Medicine, Faculty of Medicine, University of Geneva, Geneva, Switzerland

^2^ Swiss Center for Applied Human Toxicology, Geneva, Switzerland

E-mail: jean-charles.sanchez@unige.ch

**Figure S1**

**Paraquat effect on cell proliferation and cytotoxicity**

A proliferation and cytotoxicity assay were performed on PQ-treated HBMECs. Cells were exposed to PQ at 0,1, 1, 10, 100, 1000 and 5000 µM for 24 h. Results indicated a statistically significant decrease of cell proliferation after they were exposed to PQ at 1000 and 5000 µM (Fig. S1 A). HBMECs exposed to PQ at 1000 µM and 5000 µM were noticed as concentrations inducing a statistically significant cytotoxicity increase, meaning that these concentrations might lead to toxic effect in HBMEC (Fig. S1 B).

According to these results, PQ at 1, 10 and 100 µM were concentration that we kept as they are having an effect on HBMECs but with no impact on their proliferation and with no cytotoxic effect.

**A.** **B.**


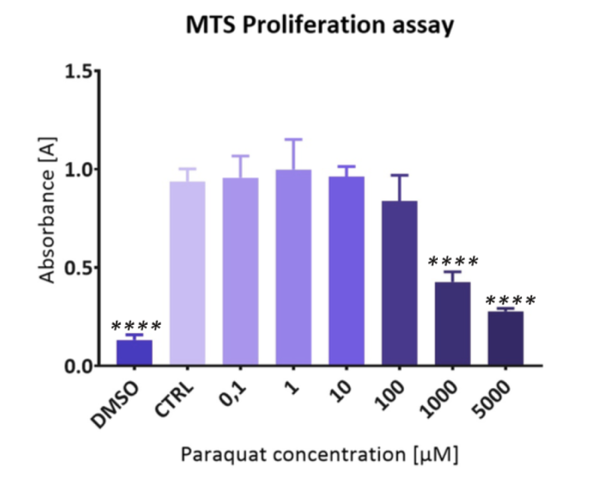
*
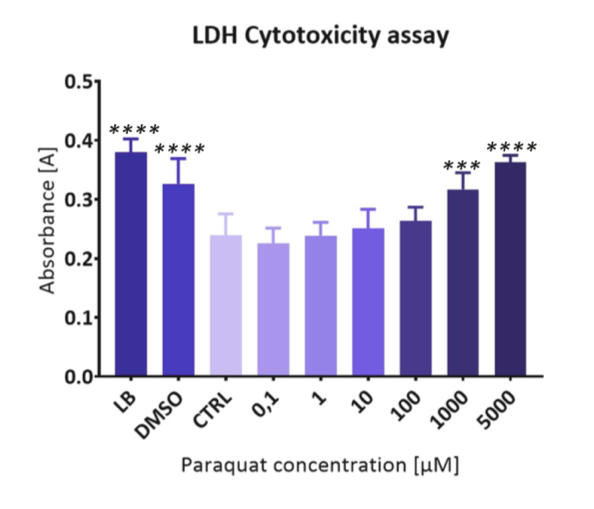
*

Fig. S1 A and B: PQ-effect on proliferation and toxicity on HBMECs after 24 h of exposition.

A. PQ toxicity assessment in HBMECs after 24 hours at different concentrations (0,1, 1, 10, 100, 1000 and 5000 µM) using MTS proliferation assay kit. B. PQ viability assessment in HBMEC after 24 hours at different concentrations (0,1, 1, 10, 100,1000 and 5000 µM) using LDH cytotoxicity assay kit. The x-axis corresponds to PQ concentration used for each assay with the controls concentration. The y-axis corresponds to the measured absorbance of formazan at 492 nm for the proliferation assay and to the measured absorbance of lactate desydrogenase at 492-680 nm for the cytotoxicity assay. Data are represented as means ±SD of three biological replicates. *** corresponds to a *p*-value ≤ 0,001 and **** corresponds to *p*-value ≤ 0,0001.
